# *Haemonchus contortus* Parasitism in Intensively Managed Cross-Limousin Beef Calves: Effects on Feed Conversion and Carcass Characteristics and Potential Associations with Climatic Conditions

**DOI:** 10.3390/pathogens11090955

**Published:** 2022-08-23

**Authors:** Konstantinos V. Arsenopoulos, Eleni I. Katsarou, Jairo A. Mendoza Roldan, George C. Fthenakis, Elias Papadopoulos

**Affiliations:** 1 Laboratory of Parasitology and Parasitic Diseases, School of Veterinary Medicine, Faculty of Health Sciences, Aristotle University of Thessaloniki, 54124 Thessaloniki, Greece; 2 Veterinary Faculty, University of Thessaly, 43100 Karditsa, Greece; 3 Department of Veterinary Medicine, University of Bari, 70010 Bari, Italy

**Keywords:** beef cattle, bodyweight, carcass quality, climate, feed conversion ratio, *Haemonchus contortus*, ivermectin, temperature-humidity index, weight gain

## Abstract

The objectives of the study were: (a) to study the effect of *Haemonchus* spp. on the growth performance characteristics of fattening calves, (b) to assess any potential effects on carcass characteristics and (c) to investigate the potential role of climatic conditions in the process of the infection. The study was conducted for 201 days in an intensively managed cross-Limousin herd. The animals were divided into two equal groups: those receiving anthelmintic treatment (AT) and the untreated (C) controls. The same nutritional regime was applied to both groups and the feed consumption was calculated daily. Standard parasitological examinations were performed at weekly intervals. At slaughter, carcasses were weighed and assessed for conformation and fat cover classes. Climatic variables were obtained for the location of the farm and a temperature-humidity index was calculated. Before anthelmintic treatment with ivermectin, there was no difference in parasitic burdens between the two groups: 544 (AT) vs. 554 (C) epg, whilst after it, counts were 0 and 450–700 epg, respectively, with over 96% of larvae identified as *Haemonchus* spp. and, later, confirmed as *Haemonchus contortus*. It was concluded that treated animals had a higher average total bodyweight gain, higher feed conversion ratio and carcass yield of superior quality than controls. There was a difference between the two groups in the pattern of reduction of dry matter intake as the temperature-humidity index increased.

## 1. Introduction

The major parasitic nematode species implicated in cattle parasitism under European management conditions are *Ostertagia* spp. and *Cooperia* spp. [1]. Nevertheless, *Haemonchus* spp. has also been identified in cattle farms [2]. Thereafter, Emery et al. [3] highlighted the cosmopolitan distribution of this nematode parasite and named it as a significant health problem for beef cattle, which has been identified in many countries throughout the world [4].

Traditionally, upon the arrival of beef cattle at a feedlot, the administration of anthelmintics is practiced, which leads to a reduction of parasitic burdens and contributes to maximizing the performance of the animals [5,6]. That way, animals have little exposure to parasitic infections within the feedlot [7]. Cases of missing anthelmintic treatments can occur, whilst the increasing prevalence of anthelmintic resistance of nematode parasites [8] may also render the anthelmintic administration ineffective. In beef cattle, internal parasitic infections have been associated mainly with decreased growth and occasionally with clinical problems, e.g., reduced appetite and diarrhoea; moreover, at herd level, increased risk of other infections has also been documented [3,7,9].

There are only limited reports available regarding the potential adverse effects of monospecific *Haemonchus* spp. parasitism on growth performance (i.e., bodyweight gain and feed conversion ratio) and carcass characteristics of beef calves [4]. Moreover, the effects of climatic conditions have not been assessed, likely because it has been postulated that in intensive management systems, these might be minimal. The objectives of the present study were (a) to study the growth performance characteristics in fattening calves parasitized with *Haemonchus* spp., using cross-Limousin as the model, (b) to assess the potential effects on carcass characteristics and (c) to investigate the potential role of climatic conditions in the process of the infection.

## 2. Results

### 2.1. Parasitological Findings

Before administration of the anthelmintic treatment, there was no difference in parasitic burdens between the two groups: 544 ± 15 epg (mean ± standard error of the mean) for anthelmintic treated (AT) group vs. 554 ± 16 epg for control (C) group (*p* > 0.45 for all comparisons). In all cases, over 97% of larvae in faecal samples from animals of the two groups were identified as *Haemonchus* spp.; moreover, *Ostertagia* spp. and *Trichostrongylus* spp. larvae were also identified in the samples.

**Figure 1:**
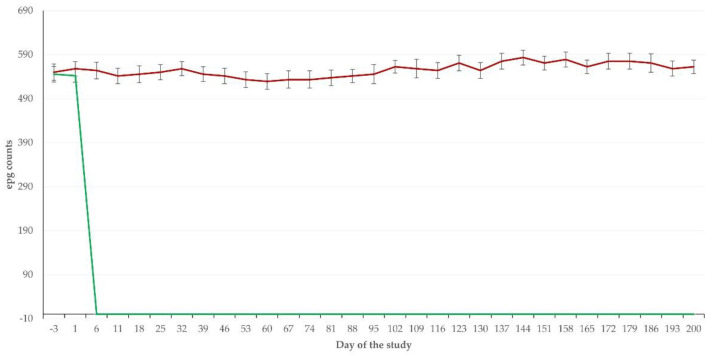
Parasitological findings (epg counts) in faecal samples from beef calves that had received anthelmintic treatment (group AT, green line) with ivermectin or were untreated controls (C, dark red line).

After administration of the anthelmintic treatment, burdens were consistently 0 epg in all animals of group AT until the end of the study. In contrast, parasitic burdens in group C were significantly higher, varying from 450 to 700 epg among individual animals and samplings throughout the study (*p* < 0.008 for all comparisons between the two groups) (Figure 1). Larvae of *Haemonchus* spp. were more frequently identified (>96%) in the faecal samples from the animals of group C; the remaining larvae were identified as *Ostertagia* spp. or *Trichostrongylus* spp.

During the post-mortem examination of the calves at slaughter, no helminths were detected in the abomasa of calves in group AT. In contrast, in the abomasa of all calves in group C, helminths macroscopically similar to *Haemonchus* spp. were found (mean number of these helminths per animal 79 ± 2, ranging from 69 to 90). During molecular assessment of each helminth collected, the Blast analysis of sequences obtained showed a high nucleotide identity ranging from 99.9% to 100% with those of *Haemonchus contortus* available in GenBank.

### 2.2. Feed Consumption and Bodyweight Gain of Calves—Feed Conversion Ratio

On D-3, there was no difference in the mean age of animals: 8.13 ± 0.09 months for calves in group AT and 8.13 ± 0.11 months for calves in group C (*p* = 0.99).

Throughout the study, calves in group AT consumed less concentrate feed and more roughage than calves in group C (*p* < 0.0001 for all comparisons). After treatment, the average daily dry matter consumption, throughout the study, was higher in calves of group C than in animals of group AT: 9.15 ± 0.06 kg vs. 8.82 ± 0.05 kg per animal, respectively (Figure S1).

Although no difference was seen in the bodyweight of animals at the start of the study (*p* = 0.83), animals of group AT were significantly heavier at the end (*p* < 0.0001); hence, weight gain was significantly higher in group AT than in group C (*p* < 0.0001). Details are in Table 1.

Based on the above, the feed conversion ratio (FCR) was calculated to be 4.96 for calves in group AT and 7.19 for calves in group C; i.e., 45% better for the former group (Figure S2).

### 2.3. Associations of Climatic Results with Parasitological Findings and Feed Consumption

The changes in the climatic parameters of the location of the farm throughout the study are presented in Figures S3–S5. There were differences in feed consumption patterns between animals in group AT and C throughout the study, in accordance with the temperature-related parameters (bar the temperature range) and the relative humidity of the farm. Details are presented in Table S1.

**Figure 2:**
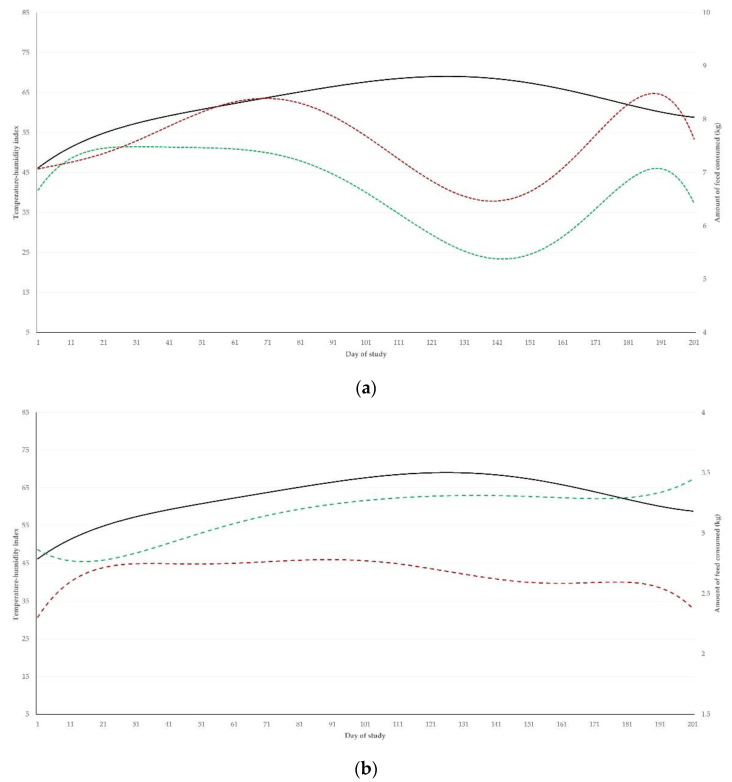
Schematic presentation of the temperature-humidity index (black solid line) throughout a study with beef calves that had received anthelmintic treatment (green dashed line) with ivermectin or were untreated controls (dark red dashed line), at the location of the farm, where the study was conducted, in association with trends of daily feed consumption of (**a**) concentrate feed or (**b**) roughage by the calves (full graph in Figure S6).

**Figure 3:**
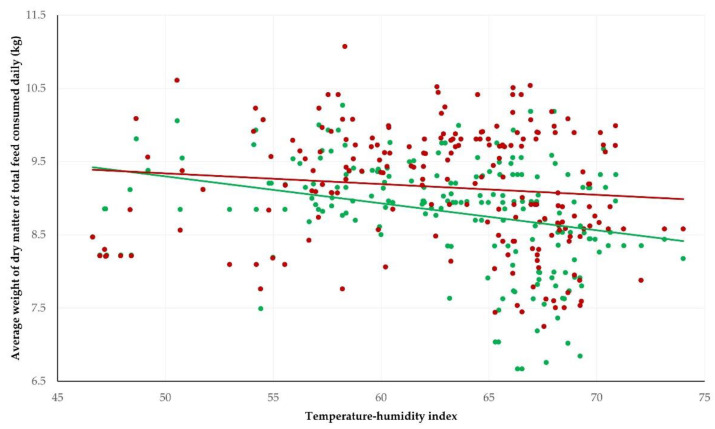
Correlations between average dry-matter intake and temperature-humidity index at the location of the farm, throughout a study with beef calves that had received anthelmintic treatment (green dots) with ivermectin or were untreated controls (dark red dots) (solid lines show tendency lines).

These differing patterns were also seen when the temperature-humidity index was applied and correlated with the feed consumption results. There was a negative difference between the correlation coefficients for the two groups for concentrate consumption and a positive difference between them for roughage consumption (Table 2 and Figure 2). This was reflected in a clear difference between the two groups in the pattern of reduction of dry matter intake as the temperature-humidity index increased: *r* = −0.302 (*p* < 0.0001) for calves in group AT and *r* = −0.109 (*p* = 0.06) for calves in group C; the difference between them was significant (*z* = −2.01, *p* = 0.022) (Figure 3).

### 2.4. Carcass-Related Parameters of Calves

Carcasses obtained by calves in group AT were heavier and produced a higher carcass yield than carcasses by calves in group C (*p* < 0.001). The combined coefficient of the conformation class and the fat cover class (Table S2) was significantly higher (on average +115%) in carcasses of animals in group AT: 0.43 ± 0.05 vs. 0.20 ± 0.03 for carcasses of animals in group C (*p* = 0.008) (Table 3, Figure S7). No relationship was seen between the number of collected helminths in the abomasa of animals and the respective carcass-related parameters (|*r*| < 0.37, *p* > 0.10 for all calculations).

## 3. Discussion

### 3.1. Parasitological Findings and Effects on Calf Production

The egg output of the parasitized group remained stable throughout the study (age at slaughter 15 months old). Though many studies record a drop of the faecal egg counts due to the acquisition of immunity against *Haemonchus* spp. [10,11,12], Urquhart et al. [13] defined that cattle over two years old are relatively immune against *Haemonchus* spp. A similar result was also recorded by George et al. [14], who identified the nematode parasite *Cooperia* spp. as the most common genus present in cattle older than 12 months, being unable to build protective immunity. It is known that rapid growth of the animals (i.e., feedlot period) is a stressful period of high nutrient demand, and therefore, the requirements of the immune system are less prioritized over the ability of the host to maintain the functions of survival and growth [15]. It is, therefore, postulated that the nutrient density (i.e., energy and protein density) of the food combined with the higher dry matter intake of the parasitized animals was insufficient to provoke a strong immune response against the parasitic burden of the animals, and therefore, they failed to reduce the FEC. Furthermore, we cannot exclude the possible reinfection of the animals, since the old construction of the farm had left areas which were difficult to clean and disinfect. The aforementioned reasons make anthelmintic treatment obligatory, at least upon the arrival of beef cattle at the feedlot.

It is generally proven that the diagnostic value of faecal egg counts (FEC) in small ruminants is greater for *H. contortus* than for other trichostrongyles, because there is a strong relationship between the total *H. contortus* count and FEC in sheep [16,17] and goats [18]. Furthermore, there is a variation in the relationship between FEC and worm counts in different hosts (i.e., sheep and goats), though in the same host, this relationship is very strong [16]. On the contrary, such relevant studies on bovine haemonchosis have not been conducted, to the best of our knowledge. Another possible explanation for the mismatch between adult *Haemonchus* helminths and the FEC in the beef calves of our study is the “virulence” of *Haemonchus* spp [3]. Virulence should refer to the relative capacity of a pathogen to cause disease, and thus, *Haemonchus contortus* virulence is expressed as increased L_3_ survival, higher L_3_ establishment rate, shorter pre-patent period and greater fecundity [19].

In a relevant study performed in the United States of America, Kunkle et al. [20] reported that parasitic burdens as low as those manifested with 140 to 270 epg in faecal samples could cause some reduction in bodyweight gain. Later, Navarre [21] indicated that gastrointestinal parasitism caused mainly subclinical infections in cattle, and thus, production effects would be more frequent than cases of morbidity in herds with increased parasitic burdens. The major nematode genera implicated in parasitic infections of cattle in Europe are *Ostertagia* spp. and *Cooperia* spp. [1,2,22,23], although *Haemonchus* spp. has also been identified as an important helminth in areas with mild climate [2,24].

In previous relevant studies, the adverse effects of various nematode genera had been assessed [25,26,27]. The present results provide the opportunity to evaluate the potential adverse effects of *Haemonchus* parasitism in beef production under natural infection circumstances. The result of 28.5% smaller bodyweight gain in infected calves is in full accord with the findings of Flores–Perez et al. [4], who, in an experimental study, indicated that bodyweight gain in parasitized calves decreased by 15% to 28%. Results of proportionate scale have been reported in cases of parasitism by *Ostertagia* spp. or *Cooperia* spp. [28,29], as well.

It is generally considered that nematode infections lead to a reduction in feed intake in ruminants [29,30]. However, Kyriazakis et al. [31], in a different approach, suggested that parasitized animals increased feed consumption in an attempt to fulfil protein requirements, with which the results of the present study are in agreement: the untreated control calves in the present study consumed more dry matter [i.e., 8.53% and 9.46% more energy (Unite Fourragere Viande, UFV) and protein (crude protein, CP), respectively, which corresponds to approximately 171 g and 187 g of bodyweight gain, respectively], compared to animals receiving the anthelmintic treatment.

Protein deficiency, and consequently, potential antibody deficiency, can be a key effect of gastrointestinal nematode infections, as described for *Teladorsagia* spp. [32] and *Haemonchus* spp. [33] infections. Nevertheless, the protein level in the feed may also play a role in the development and enhancement of resistance to parasitic infections [34]: parasitized sheep provided with a feed with high-protein content were better able to balance an infection by *H. contortus* compared to animals fed with a standard diet, as indicated by lower faecal epg counts and smaller helminth burdens. Thus, possibly, the parasitized calves increased their dry matter intake to balance this protein deficit, thereby also enhancing their immune status.

During the study, the observed lower feed conversion ratio, which has a direct financial significance for farmers, can be a reflection of the combined effects of the reduced average daily weight gain and the increased consumption of dry matter by the parasitized calves, as indicated above. In a similar study performed in lambs, Kyriazakis et al. [31] reported a reduction in feed conversion ratio of 15% to 24% in feedlot lambs parasitized by *Trichostrongylus colubriformis*.

### 3.2. Associations of Climatic Results with Parasitological Findings and Feed Consumption

Ruminants, like other mammals, dissipate heat by evaporation, radiation, convection and conduction according to endogenous homeostatic regulations aiming to maintain a constant internal temperature, at 39 °C [35]. Within the zone of thermoneutrality, between the lower and upper critical temperatures, heat production is dependent on body weight, energy intake, digestive work (i.e., fermentations, gut motricity, chewing, etc.) and metabolic inefficiency. When environmental temperature increases above the upper critical value, thermoregulatory mechanisms progressively promote heat production through convection and evaporation from skin by means of subcutaneous vasodilatation, sweating and through the lungs by increasing the respiration rate. Simultaneously, dry matter intake normally decreases, as feed consumption is an important cause of heat production [36]. Further, the rate of evaporation by an animal is also linked to environmental humidity. Thus, at a given temperature, increasing humidity has a more marked impact on animal feed intake and performance [35]. Heat stress can be best described by the temperature-humidity index, which has been accepted for the quantification of its occurrence and severity. In the present study, an index that has been found to be specific for use in studies related to fattening calves was used, as these animals are considered to have a narrow thermoneutral zone [37]. Although several studies have presented the effects of heat stress on dairy cows, such potential effects on beef calves have not been widely evaluated [38].

Animals in both groups showed a decrease in dry matter intake when the temperature-humidity index increased over 65, which indicates the start of heat stress conditions. Nevertheless, this reduction was significantly smaller among untreated controls than those that had received anthelmintic treatment.

Studies on the behavioural patterns of cattle, in accord with changes in the temperature-humidity index, have indicated that heat stress may lead to an increase of time spent in the standing position [39]. Spending time in the standing position may permit cattle to maximize the effective surface area for heat release from body surfaces, to reduce heat from the warm ground and to increase the efficiency of respiration [40]. However, this would contribute to increased muscular effort by the animals, which would further contribute to increased energy requirements. Thus, one may postulate that animals reacted to this increased energy deficit (parasitic infection plus muscular effort) by significantly increasing dry-matter intake in order to fulfil the increased energy demands [41] that resulted from the combined parasitism and muscular effort.

### 3.3. Carcass-Related Parameters of Beef Calves

The potential effects of *Haemonchus* parasitism on carcass-related parameters of beef calves has not been fully documented in the literature. The smaller carcass yield of the untreated animals is the result of the lower feed conversion ratio and reduced growth rate of these calves. It is generally accepted that heavily parasitized cattle take a longer time to reach a market-ready carcass [7]. For this reason, the combined evaluation of the conformation and the fat cover, using the European score system of carcasses (Regulation EU No 1308/2013) was employed. The findings indicated an improved muscle conformation with smaller fat cover among treated animals, which led to inferior body conformation. The infected calves produced carcasses with more fat, which can be associated with the increased consumption of concentrates and reduced consumption of roughage. This feeding regime leads to production of higher propionic acid/lower acetic acid in the rumen, which constitute glycogen precursors and participate in the formation of fatty acids of the adipose tissue. Moreover, one can postulate that availability of nutrients relevant for muscular growth and development was reduced, as these were uptaken by parasites [7]; another potential explanation might be the redirection of nutrients for an immune response against the parasitising helminths.

## 4. Materials and Methods

### 4.1. Experimental Design and Study Protocols

The study was conducted during the period of April to October 2020, for a total of 201 days. It was conducted at a commercial farm with intensively managed cross-Limousine beef bull calves, located in Central Greece. The farm was selected for the study based on convenience, i.e., the willingness of the farmer to collaborate and receive regular visits for examination and sampling of animals in the farm.

The animals were purchased by the farmer for commercial purposes and were brought into the farm at the age of approximately 8 months, immediately after weaning, from various farms in the same area. Before arrival at the farm and inclusion into the study, no anthelmintic treatment had been implicated to the animals for at least four months.

Animals arrived at the farm and were penned together for an accustomization period of 4 days. Then, they were allocated into two equal groups (*n* = 12) on D1. Allocation concealment was performed. A randomization schedule in a 1:1 ratio was performed (author E.P.) for allocation of calves in groups: AT (for anthelmintic treatment) or C (controls). The investigator who administered the anthelmintic (author K.V.A.), had not been aware in advance of the treatment for each new animal that he handled; group allocation of calves was announced to him after he had finished the clinical examination and the weighing of each animal and had prepared it for anthelmintic or placebo administration. Animals in group AT were administered ivermectin (VALANEQ^®^; Gerolymatos, Athens, Greece) subcutaneously, at a dose rate of 0.2 mg per kg bodyweight. Animals in group C received a subcutaneous injection of normal saline at a dose rate of 1 mL per 50 kg bodyweight.

After allocation into groups, all animals of each group were introduced into clean and disinfected pens with concrete floor, in an old-constructed building (Figure S8). These farms are considered typical for the fattening period of the intensively managed beef calves in Greek territory. Animals of the same group were penned together into a large pen and separately from those of the other group, with a distance of 20 m between the two pens. According to the routine of the farm, the cleaning process was performed on weekly intervals. Calves were identified using neck straps and plastic ear tags with unique farm-specific serial numbers. The farmer and farm personnel were not aware of the treatment status of the animals.

The same nutritional regime was applied to both groups throughout the study. It included a commercially prepared concentrate feed and wheat straw (Table S3). All feed were offered to the animals *ad libitum* and at group basis. The amount of feed (concentrate feed and roughage) offered to the animals and the amount that was not consumed by the animals (i.e., those that remained in the troughs), were measured on group basis every 24 h, throughout the study.

The vaccination schedule included the administration of a combined vaccine preparation against *Bovine respiratory syncytial virus*, and *Manheimia haemolytica* (Bovilis Bovipast^®^ RSP; MSD Animal Health, Kenilworth, NJ, USA). All animals were administered the first dose of the vaccine on D7 (i.e., 10 days after arrival at the farm), which was followed by a booster dose 30 days later.

Finally, on D201, animals were weighed again and taken to the abattoir. After slaughter (Figure S9), carcass of each animal was weighed and scored according to the European Union scale for the classification of carcasses of bovines for conformation class and fat cover class.

### 4.2. Parasitological Examinations

Faecal samples were collected on the day of arrival of the animals at the farm, on D1, on D6, on D11 and thereafter at weekly intervals (D18, D25, …, D193 and D200), i.e., 2 samples were collected before treatment administration and 29 samples after that. On each sampling occasion, samples were collected from each animal individually.

Samples were collected directly from the rectum of each animal, put into plastic bags, labeled, and stored in isothermal boxes for immediate transport to the laboratory. Transport of samples to the laboratory was made by car by the investigators. Their processing started within 5 to 6 h after collection.

Standard parasitological techniques were performed on the faecal samples, specifically the McMaster technique, the flotation method, the sedimentation technique and coproculture [42]. Each of the first three techniques were applied twice in each individual sample, whilst coproculture was performed once. Larvae recovered after coproculture, were identified at genus level by using the criteria of Van Wyk and Mayhew [43].

At slaughter of animals, helminths macroscopically similar to *Haemonchus* spp. (Figure S10) were collected into vials with ethanol 99%. Effort was put to collect as many worms as possible, given the difficulties of this procedure under the abattoir’s routine. Therefore, we cannot exclude the possibility of failure to collect all worms, as our scope was to collect them for molecular analysis and not to estimate the parasitic burden. Transport to the laboratory was made as above, and upon arrival, they were stored at 4 °C until processing. The head of each individual helminth was dissected from the rest of the body at the cervical papillae, excluding the parasite’s uterus and eggs, and was taken for DNA extraction. Whole genomic DNA was extracted using the protocol of Hillis et al. [44]. For species identification, a fragment of 321 base pairs of the internal transcribed spacer 2 (ITS_2_) gene of nuclear DNA (Figure S11) was amplified using a pair of primers (Table S4) described by Brasil et al. [45], produced by Eurofins Genomics GmbH (Ebersberg, Germany).

### 4.3. Data Management and Analysis

Data were entered into Microsoft Excel and analysed using SPSS v. 21 (IBM Analytics, Armonk, NY, USA). Basic descriptive analysis was performed.

Comparison of parasitological findings (epg counts) between groups AT and C was initially performed by means of analysis of variance for samples collected before administration of anthelmintic treatment (D-3, D1).

Daily consumption of concentrate feed and roughage was calculated separately for each group of animals every 24 h; for this, the amount of respective feed (concentrate or roughage) that was not consumed and remained in the troughs at the end of the 24 h period was subtracted from the amount of feed offered to animals during the same period. Weight gain was calculated as the difference in bodyweight at slaughter compared to the bodyweight on D1. Based on the above data, feed conversion ratio was calculated as the weight of the dry matter of total feed consumed by all animals of each group throughout the study divided by the total weight gain of all animals in that group. For this calculation, dry matter was taken to be 87.65% for the concentrate feed (as indicated by the manufacturer) and 93.60% for wheat straw [46]. Data on feed consumption, bodyweight and weight gain were compared between groups by employing analysis of variance.

Data on the location of the farm were collected using hand-held Global Positioning System Garmin units. The geo-references were resolved to the specific farm level. Climatic variables were derived from ‘The POWER (Prediction of Worldwide Energy Resources) Project’ (NASA Langley Research Center (LaRC), Hampton, VA, USA), which provides meteorological datasets from NASA research for the support of agricultural needs. The following settings were used for obtaining the data: user community, ‘*agroclimatology*’; temporal average, ‘*daily*; latitude/longitude, ‘*geo-references the farm*’; time extent, ‘*start date 1st April 2020–18th October 2020′*; output file format, ‘*ASCII*’. Daily data for the following parameters were extracted: mean temperature at 2 m (T2M), temperature of Earth skin (TS), minimum temperature at 2 m (T2Min), maximum temperature at 2 m (T2Max), temperature range at 2 m (T2Ran), relative humidity at 2 m (RH2m) and precipitation (PREC). Based on the mean temperature at 2 m and the relative humidity at 2 m, obtained as above, the ‘temperature-humidity index’ (THI) described by Berman et al. [47] and de Oliveira Nascimento et al. [48] for housed cattle was calculated daily throughout the study, as follows: THI = 3.43 + (1.058 × T2M) − (0.293 × RH2m) + (0.0164 × T2M × RH2m) + 35.70. Subsequently, the potential associations of each of the climatic parameters and of the temperature-humidity index with the consumption of concentrate feed and roughage were assessed on daily basis (*n* = 201) and separately for each of the two groups by using analysis of correlation. Then, by employing the Fisher *r*-to-*z* transformation, a value of *z* that could be applied to evaluate the significance of the difference between the correlation coefficients found for respective comparisons between the two groups of animals, was calculated.

Carcass yield was calculated as the weight of the carcass of an animal divided by the bodyweight of the animal on D201. At slaughter, carcasses were scored as S (superior), E (excellent), U (very good), R (good), O (fair) or P (poor) in accord with their conformation class (Table S5); they were also scored on a 1 (low) to 5 (very high) scale in accord with the fat cover class (Table S5). Subsequently, each conformation class and fat cover class were assigned a value from 0.0 to 1.0 (0.20 and 0.25 steps, respectively) (Table S5); finally, these two values were multiplied between them to obtain, for each carcass, a combined coefficient that took into account both carcass evaluation parameters. Data on carcass-related parameters were compared between groups by employing analysis of variance. Analysis of correlation was used to assess potential relationships between the number of *Haemonchus* parasites recovered from untreated calves during post-mortem examination with the various carcass-related parameters.

In all analyses, statistical significance was defined at *p* < 0.05.

## 5. Conclusions

The results of the present study indicated that *H. contortus*-infected calves had significantly lower production (bodyweight gain and feed conversion ratio) and yielded lighter carcasses of inferior quality than animals which were parasite-free. Dry matter intake was reduced as the temperature-humidity index increased; dry-matter intake remained higher among parasitized animals compared to non-parasitised ones.

## Figures and Tables

**Table 1:** Details of feed consumption and bodyweight of beef calves that had received anthelmintic treatment (group AT) with ivermectin or were untreated controls (C).

Group	Average Daily Feed Consumption per Group of Animals (kg)		Mean Bodyweight per Group of Animals (kg)		Average Total Bodyweight Gain per Group of Animals (kg)
	Concentrate feed	Roughage	on D1	on D201	
AT (*n* = 12)	80.4 ± 0.7	37.9 ± 0.2	214.3 ± 5.4	569.8 ± 7.9 ^1^	355.5 ± 5.7 ^1^
C (*n* = 12)	91.0 ± 0.7	32.0 ± 0.2	212.8 ± 4.2	467.3 ± 7.4 ^1^	254.5 ± 4.1 ^1^

^1^*p* < 0.0001 between groups.

**Table 2:** Correlations between temperature-humidity index and feed consumption assessed on a daily basis (*n* = 201 days) for beef calves that had received anthelmintic treatment (group AT) with ivermectin or were untreated controls (C).

Correlations for Consumption of Concentrate Feed (*r*)			Correlations for Consumption of Roughage (*r*)		
Group AT (*r*)	Group C (*r*)	Difference (*z*)	Group C (*r*)	Group AT (*r*)	Difference (*z*)
−0.471	−0.138	−3.71	0.543	0.108	4.97
*p* < 0.0001	*p* = 0.026	*p* = 0.0001	*p* < 0.0001	*p* = 0.06	*p* < 0.0001

**Table 3:** Results of evaluation of carcass-related parameters for beef calves that had received anthelmintic treatment (group AT) with ivermectin or were untreated controls (C) (*p* < 0.001 for all comparisons between groups).

Group	Mean Carcass Weight per Group of Animals (kg)	Mean Carcass Yield per Group of Animals (%)	Median Carcass Conformation Class per Group of Animals	Median Carcass Fat Cover Class per Group of Animals	Mean Coefficient for Carcass Conformation Class and Fat Cover Class per Group of Animals
AT (*n* = 12)	350.5 ± 4.3	61.5% ± 0.2%	U (min: R–max: E)	2 (min: 4–max: 2)	0.43 ± 0.05
C (*n* = 12)	262.1 ± 4.2	56.1% ± 0.3%	R (min: O–max: U)	3 (min: 4–max: 2)	0.20 ± 0.03

## Data Availability

Most data presented in this study are in the main text or in the Supplementary Materials.

## Supplementary Materials

The following supporting information can be downloaded at: https://www.mdpi.com/article/10.3390/pathogens11090955/s1, Figure S1: Dry-matter consumption by beef calves that had received anthelmintic treatment or were untreated controls; Figure S2: Feed conversion ratio of beef calves that had received anthelmintic treatment or were untreated controls, from the age of approximately 8 months and for 201 days; Figure S3: Temperature-related parameters that prevailed throughout a study with beef calves that had received anthelmintic treatment or were untreated controls, at the location of the farm, where the study was conducted; Figure S4: Precipitation and relative humidity that prevailed throughout the study at the location of the farm, where the study was conducted; Figure S5: Temperature-humidity index throughout the study at the location of the farm, where the study was conducted; Table S1: Correlations between climatic parameters and feed consumption assessed on daily basis for beef calves that had received anthelmintic treatment or were untreated controls; Figure S6: Temperature-humidity index throughout a study with beef calves that had received anthelmintic treatment or were untreated controls, at the location of the farm, where the study was conducted, in association with daily feed consumption of (a) concentrate feed or (b) roughage by the calves; Table S2: Detailed results of scoring of the carcasses of beef calves that had received anthelmintic treatment or were untreated controls; Figure S7: Mean combined coefficient for conformation class and fat cover class of carcasses of beef calves that had received anthelmintic treatment or were untreated controls; Figure S8: Concrete floor in the pen of cross-Limousin beef calves of the old-constructed farm; Table S3: Analysis of the commercially manufactured concentrate feed that was provided to beef calves; Figure S9: The beef calf after slaughter; Figure S10: Post-mortem examination of a calf’s abomasum; Table S4: Primers used and the product size, after the amplification of the internal transcribed spacer 2 (ITS_2_) of nuclear DNA of *Haemonchus* spp.; Table S5: Scoring system used for the classification of the carcasses of calves (Regulation (EU) No 1308/2013); Figure S11: Detection of the amplified internal transcribed spacer 2 (ITS_2_) of *Haemonchus* spp.
