# Supplementary material for: Haemonchus contortus Parasitism in Intensively Managed Cross-Limousin Beef Calves: Effects on Feed Conversion and Carcass Characteristics and Potential Associations with Climatic Conditions
Source: Pathogens. 2022 Aug 23;11(9):955. doi: 10.3390/pathogens11090955 (PMC9500760; doi:10.3390/pathogens11090955)
Supplement: Supplementary file 1 [file pathogens-11-00955-s001.zip › pathogens-1863624-supplementary.pdf]

Haemonchus contortus Parasitism in Intensively Managed Cross-Limousin Beef Calves: Effects on Feed Conversion and Carcass Characteristics and Potential Associations with Climatic Conditions

Konstantinos V. Arsenopoulos, Eleni I. Katsarou, Jairo A. Mendoza Roldan, George C. Fthenakis, Elias Papadopoulos

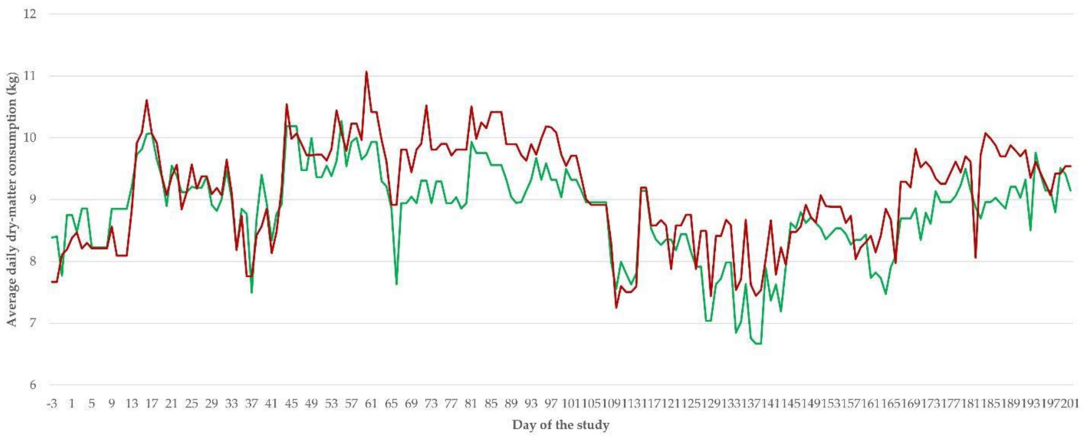

Figure S1. Dry-matter consumption by beef calves that had received anthelmintic treatment (green line) with ivermectin or were untreated controls (dark red line).
Green line: calves with anthelmintic treatment, dark red line: untreated control animals.
Average dry-matter consumption; before treatment :8.33 ± 0.20 kg per animal (calves to be treated) versus 7.91 ± 0.14 kg per animal (control calves) (p = 0.14), after treatment: 8.82 ± 0.05 kg per animal (treated calves) versus 9.15 ± 0.06 kg per animal (control calves) (p = 0.0003), slope: –0.0035 ± 0.0008 (treated calves) versus –0.0006 ± 0.0010 (control calves) (p = 0.0024).
Time frame of the study: D-3: arrival of animals at the farm, D0: anthelmintic treatment, D201: end of the study.

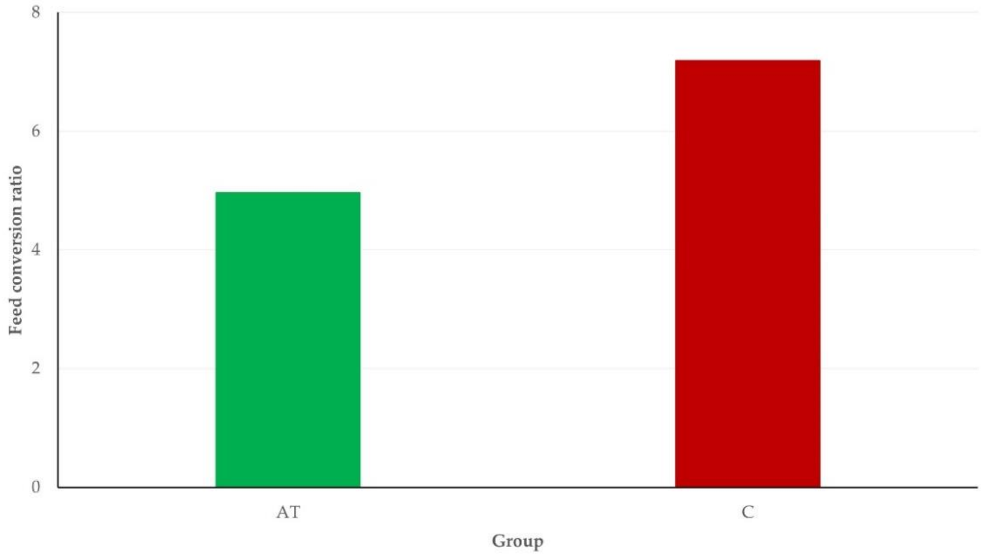

Figure S2. Feed conversion ratio of beef calves that had received anthelmintic treatment (group AT, green bar) with ivermectin or were untreated controls (group C, dark red bar), from the age of approximately 8 months and for 201 days.

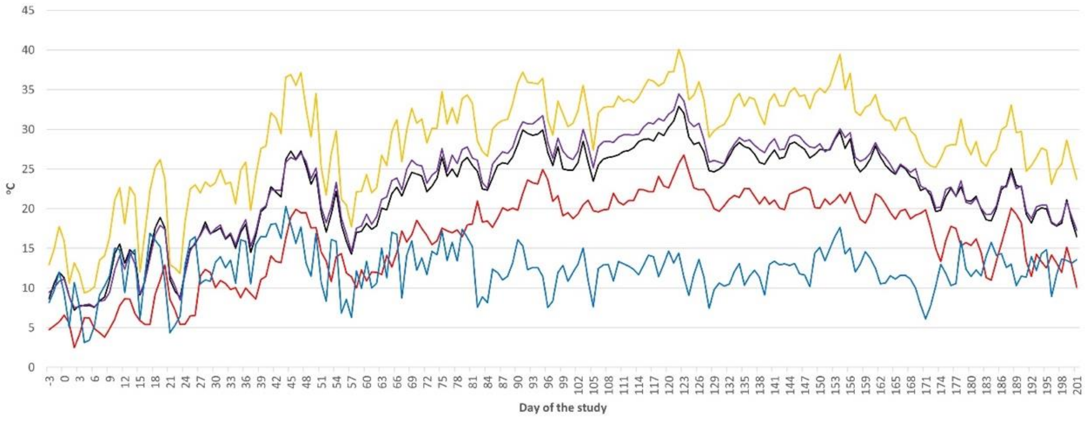

Figure S3. Temperature-related parameters that prevailed throughout a study with beef calves that had received anthelmintic treatment with ivermectin or were untreated controls, in the location of the farm, where the study was conducted.

Black line: mean temperature at 2 m; violet line: temperature of Earth skin; red line: minimum temperature at 2 m; yellow line: maximum temperature at 2 m; blue line: temperature range at 2 m. Time frame of the study: D1: 1st April 2020; D201: 18th October 2020.

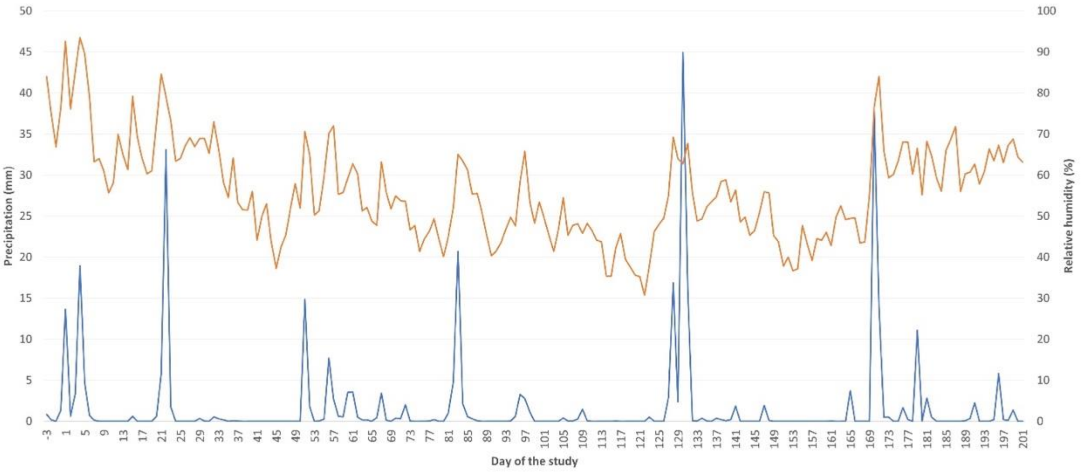

Figure S4. Precipitation and relative humidity that prevailed throughout the study in the location of the farm, where the study was conducted.
Blue line: precipitation; brown line: relative humidity.
Time frame of the study: D1: 1st April 2020; D201: 18th October 2020.

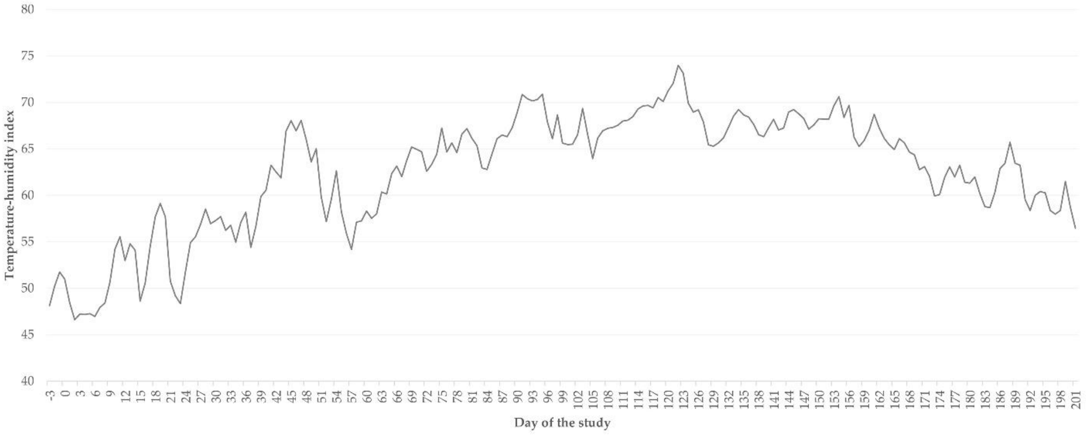

Figure S5. Temperature-humidity index throughout the study at the location of the farm, where the study was conducted.
Temperature-humidity index (THI) calculated as follows:
THI = 3.43 + (1.058 × T2M) – (0.293 × RH2m) + (0.0164 × T2M × RH2m) + 35.70, where T2M: mean temperature at 2 m and RH2M: mean temperature at 2 m. Time frame of the study: D1: 1st April 2020; D201: 18th October 2020.

Table S1. Correlations between climatic parameters and feed consumption assessed on daily basis (n = 201) for beef calves that had received anthelmintic treatment (group AT) with ivermectin or were untreated controls (C).

Climatic parameter	**Correlation coefficients for consumption of concentrate feed (r)**			**Correlation coefficients for consumption of roughage (r)**		
	Group AT (r)	Group C (r)	Difference between groups (z)	Group AT (r)	Group C (r)	Difference between groups (z)
Temperature at 2 m	–0.47124*	–0.13809*	–3.71*	0.54356*	0.10937*	4.98*

Temperature of Earth skin	−0.47431*	−0.14448*	−3.68*	0.53431*	0.12237*	4.71*
Minimum temperature at 2 m	−0.52085*	−0.16874*	−4.05*	0.56297*	0.10075	5.33*
Maximum temperature at 2 m	−0.41615*	−0.10102	−3.40*	0.51361*	0.07927	4.87*
Temperature range at 2 m	0.05573	0.09182	−0.36	0.07888	−0.0133	0.18
Relative humidity at 2 m	0.30715*	0.13739*	1.78*	−0.37732*	−0.04860	−3.46*
Precipitation	−0.02205	−0.01495	−0.07	−0.04755	0.13070*	−1.78*

*: $p < 0.05$.

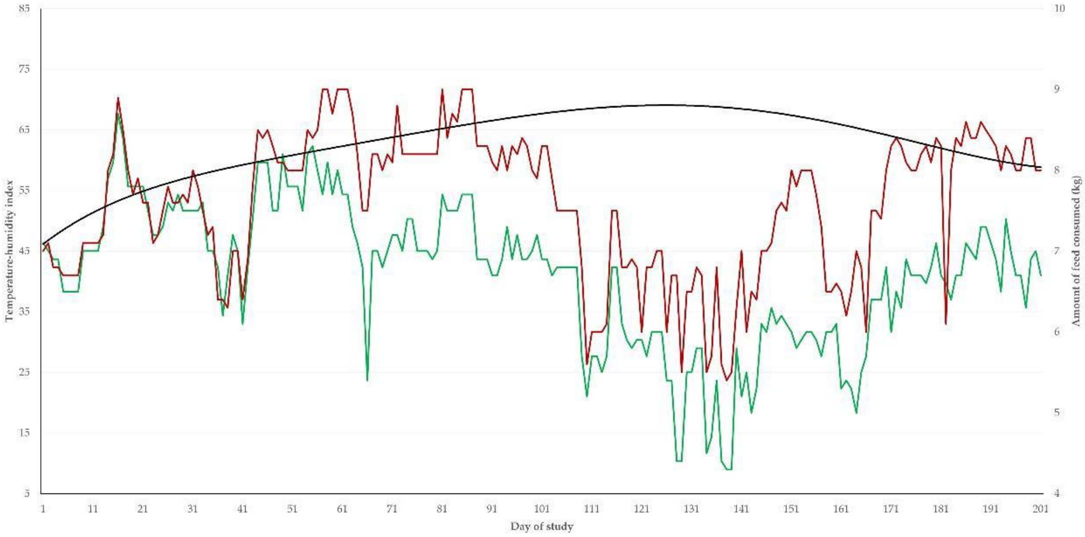

(a) Consumption of concentrate feed

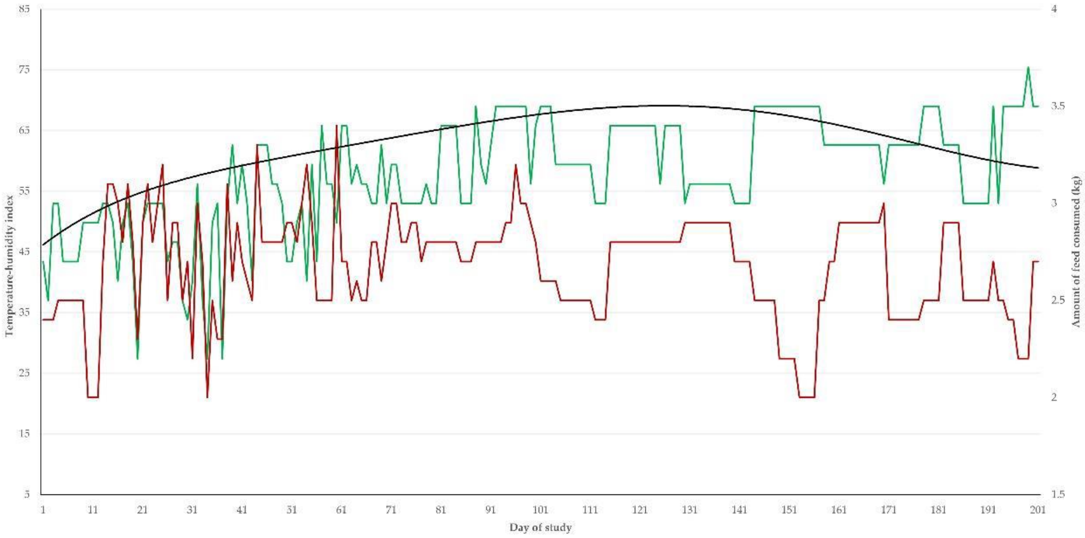

(b) Consumption of roughage

Figure S6. Temperature-humidity index (black solid line) throughout a study with beef calves that had received anthelmintic treatment (green line) with ivermectin or were untreated controls (dark red line), at the location of the farm, where the study was conducted, in association with daily feed consumption of (a) concentrate feed or (b) roughage by the calves.
Temperature-humidity index (THI) calculated as follows:
THI = 3.43 + (1.058 × T2M) – (0.293 × RH2m) + (0.0164 × T2M × RH2m) + 35.70, where T2M: mean temperature at 2 m and RH2M: mean temperature at 2 m. Time frame of the study: D1: 1st April 2020; D201: 18th October 2020.

Table S2. Detailed results of scoring of the carcasses of beef calves that had received anthelmintic treatment (group AT) with ivermectin or were untreated controls (C).

**Group AT**						**Group C**					
Animal no.	Conforma-tion class	Value assigned	Fat cover class	Value assigned	Combined coefficient	Animal no.	Conforma-tion class	Value assigned	Fat cover class	Value assigned	Combined coefficient[1]
1	U	0.60	2	0.75	0.45	13	R	0.40	3	0.50	0.20
2	E	0.80	2	0.75	0.60	14	R	0.40	3	0.50	0.20
3	R	0.40	4	0.25	0.10	15	O	0.20	4	0.25	0.05
4	U	0.60	2	0.75	0.45	16	O	0.20	4	0.25	0.05
5	E	0.80	2	0.75	0.60	17	R	0.40	3	0.50	0.20
6	U	0.60	3	0.50	0.30	18	O	0.20	3	0.50	0.10
7	U	0.60	3	0.50	0.30	19	U	0.60	3	0.50	0.30
8	U	0.60	2	0.75	0.45	20	U	0.60	3	0.50	0.30
9	E	0.80	2	0.75	0.60	21	R	0.40	3	0.50	0.20
10	E	0.80	2	0.75	0.60	22	R	0.40	4	0.25	0.10
11	U	0.60	2	0.75	0.45	23	U	0.60	2	0.75	0.45
12	R	0.40	3	0.50	0.20	24	R	0.40	3	0.50	0.20

[1]: combined coefficient calculated as the product of the value assigned to conformation class by the value assigned to fat cover class.

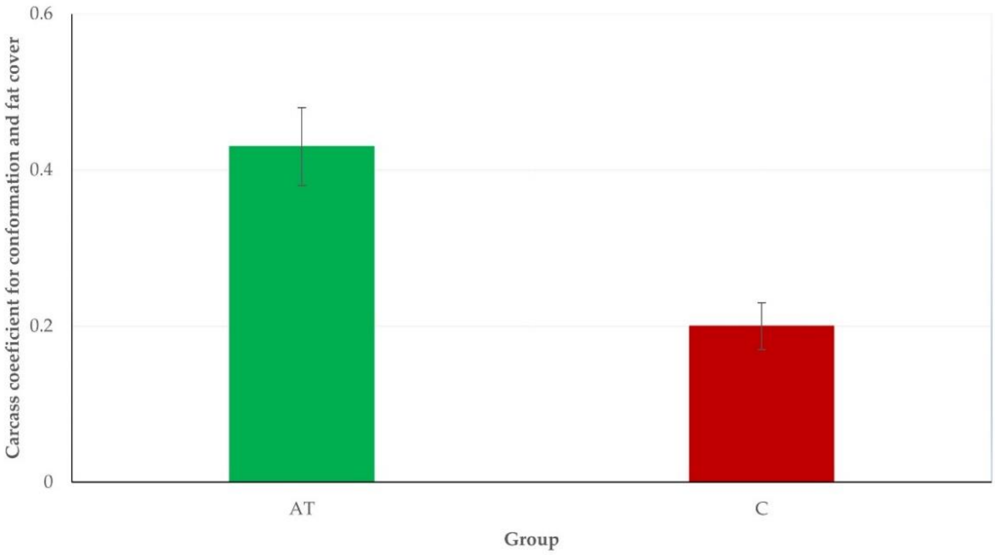

Figure S7. Mean combined coefficient for conformation class and fat cover class of carcasses of beef calves that had received anthelmintic treatment (group AT, green bar) with ivermectin or were untreated controls (group C, dark red bar).

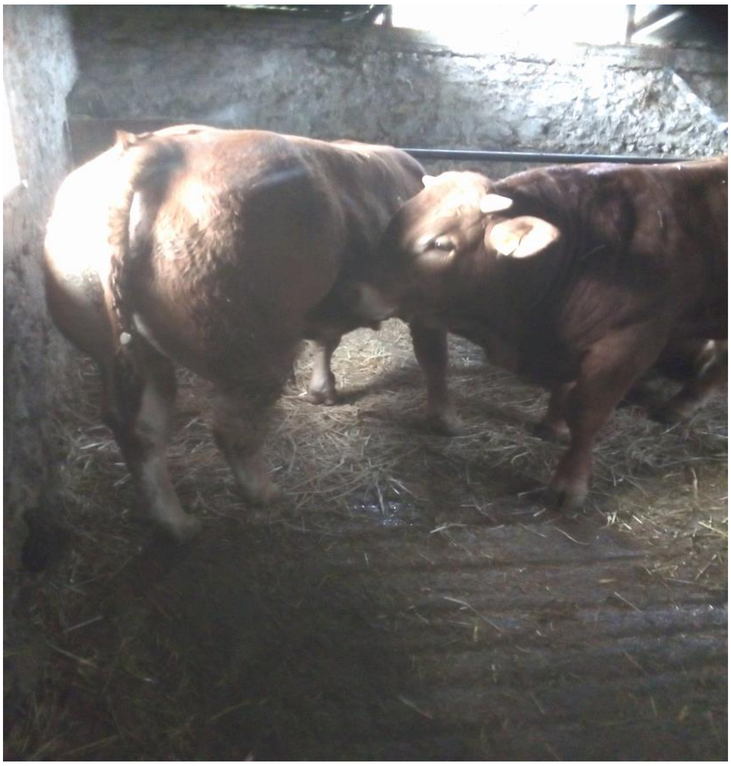

Figure S8. Concrete floor in the pen of cross-Limousin beef calves of the old-constructed farm. The wheat straw was used as a bedding material.

Table S3. Analysis of the commercially manufactured concentrate feed that was provided to beef calves.

Ingredient	Proportion of inclusion (%)
Corn meal	50.0
Barley meal	12.0
Soyabean meal (47.0% crude protein)	14.5
Molasse	3.0
Beet pulp	13.0
Hydrogenated fat	2.0
Urea	0.5
Premix	2.5
Sodium chloride	1.0
Sodium bicarbonate	1.5
**Nutrient**	**Value**
Dry matter	87.65%
Unité Fourragère Viande (UFV)	114.63%
Protein digestible (PDIN)	11.99%
PDIN/UFV	101.55
Protein digestible (PDIE)	11.39%
PDIE/UFV	99.40
Crude protein	16.67%
Crude fat	4.69%
Starch	43.71%
Sugars	5.06%
Neutral detergent fibre (NDF)	16.52%
Acid detergent fibre (ADF)	6.50%

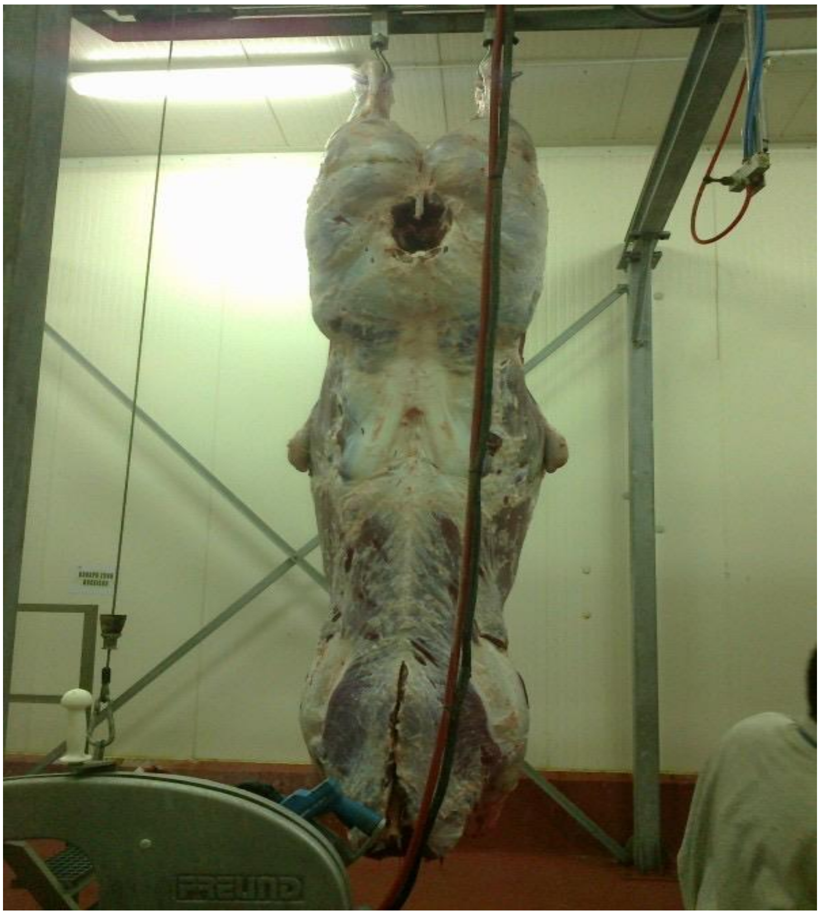

Figure S9. The beef calf after slaughter

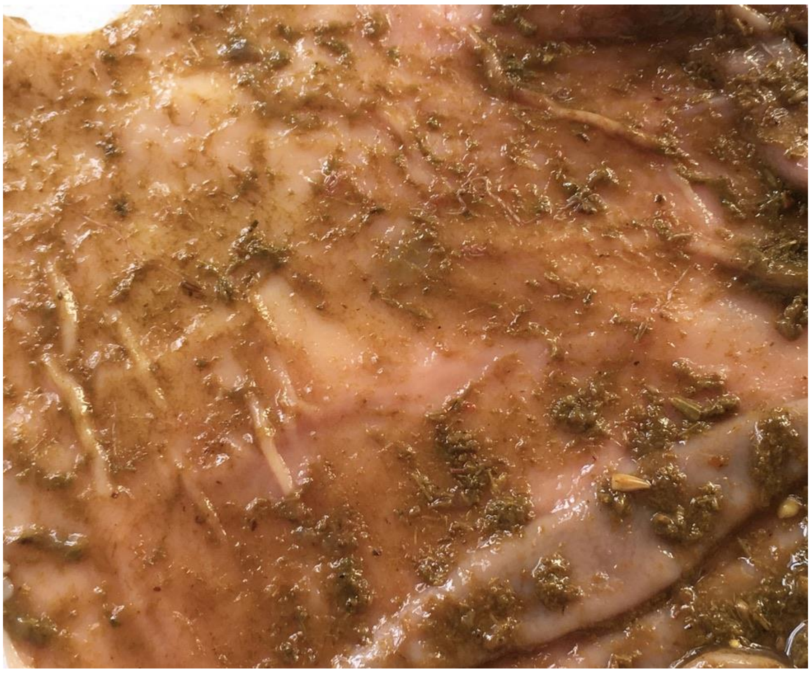

Figure S10. *Haemonchus* spp. on a calf's abomasum

Table S4. Primers used and the product size, after the amplification of the internal transcribed spacer 2 (ITS_2) of nuclear DNA, of *Haemonchus* spp.

Primer	Sequence	Product Size (base pairs)
NC1-F	forward: 5´- ACGTCTGGTTCAGGGTTGTT -3´	321
NC2-R	reverse: 5'- TTAGTTTCTTTTCCTCCGCT -3´	

Table S5. Scoring system used for the classification of the carcasses of calves [Regulation (EU) No 1308/2013].

(a) Conformation class

Conformation class	Description	Value assigned for analysis of results
S (Superior)	All profiles extremely convex; exceptional muscle development (double muscled carcass type)	1.00
E (Excellent)	All profiles convex to super-convex; exceptional muscle development	0.80
U (Very good)	Profiles on the whole convex, very good muscle development	0.60
R (Good)	Profiles on the whole straight; good muscle development	0.40
O (Fair)	Profiles straight to concave; average muscle development	0.20
P (Poor)	All profiles concave to very concave; poor muscle development	0.00

(b) Fat cover class

Fat cover class	Description	Value assigned for analysis of results
1 (Low)	None up to low fat cover	1.00
2 (Slight)	Slight fat cover, flesh visible almost everywhere	0.75
3 (Average)	Flesh with the exception of the round and shoulder, almost everywhere covered with fat, slight deposits of fat in the thoracic cavity	0.50
4 (High)	Flesh covered with fat, but on the round and shoulder still partly visible, some distinctive fat deposits in the thoracic cavity	0.25
5 (Very high)	Entire carcass covered with fat; heavy deposits in the thoracic cavity	0.00

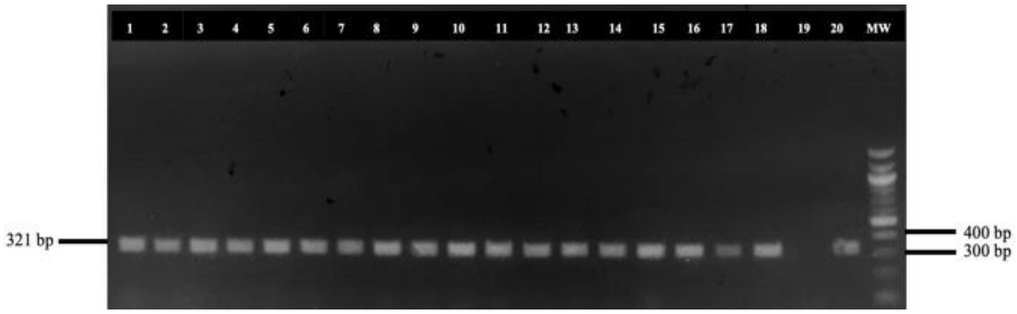

Figure S11. Detection of the amplified internal transcribed spacer 2 (ITS$_2$) of *Haemonchus* spp. MW: 100 base pairs ladder, 1-18 and 20: amplified ITS$_2$ of collected *Haemonchus* spp., 19: negative control sample
